# Sagittal and transverse ankle angle coupling can influence prosthetic socket transverse plane moments

**DOI:** 10.3389/fresc.2024.1354144

**Published:** 2024-04-04

**Authors:** Glenn K. Klute, Connor W. Mulcahy

**Affiliations:** ^1^ US Department of Veterans Affairs, Center for Limb Loss and MoBility, Seattle, WA, United States; ^2^ Department of Mechanical Engineering, University of Washington, Seattle, WA, United States

**Keywords:** prosthesis, lower limb, amputation, residual limb, torsion adapter, transverse plane rotation adapter

## Abstract

**Introduction:**

The intact foot and ankle comprise a complex set of joints that allow rotation in multiple planes of motion. Some of these motions are coupled, meaning rotation in one plane induces motion in another. One such coupling is between the sagittal and transverse planes. For every step, plantar- and dorsi-flexion motion is coupled with external and internal rotation of the shank relative to the foot, respectively. There is no prosthetic foot available for prescription that mimics this natural coupling. The purpose of this study was to determine if a sagittal:transverse ankle angle coupling ratio exists that minimizes the peak transverse plane moment during prosthetic limb stance.

**Methods:**

A novel, torsionally active prosthesis (TAP) was used to couple sagittal and transverse plane motions using a 60-watt motor. An embedded controller generated transverse plane rotation trajectories proportional to sagittal plane ankle angles corresponding to sagittal:transverse coupling ratios of 1:0 (rigid coupling analogous to the standard-of-care), 6:1, 4:1, 3:1, and 2:1. Individuals with unilateral transtibial amputation were block randomized to walk in a straight line and in both directions around a 2 m circle at their self-selected speed with the TAP set at randomized coupling ratios. The primary outcome was the peak transverse plane moment, normalized to body mass, during prosthetic limb stance. Secondary outcomes included gait biomechanic metrics and a measure of satisfaction.

**Results:**

Eleven individuals with unilateral transtibial amputations participated in the study. The 6:1 coupling ratio resulted in reduced peak transverse plane moments in pairwise comparisons with 3:1 and 2:1 coupling ratios while walking in a straight line and with the prosthesis on the outside of the circle (*p* < .05). Coupling ratio had no effect on gait biomechanic metrics or satisfaction.

**Discussion:**

The general pattern of results suggests a quadratic relationship between the peak transverse plane moment and coupling ratio with a minimum at the 6:1 coupling ratio. The coupling ratio did not appear to adversely affect propulsion or body support. Subjects indicated they found all coupling ratios to be comfortable. While a mechatronic prosthesis like the TAP may have limited commercial potential, our future work includes testing a robust, passive prosthetic foot with a fixed coupling ratio.

## 1 Introduction

Ambulatory individuals with a lower limb amputation are prone to pain and injury caused by loads applied to the residual limb through the prosthetic socket (1–5). Epidermoid cysts, for example, are painful lesions of the residual limb caused by shear stress where the skin of the residual limb rubs against the brim of the socket (6). The high stress at the prosthesis-residual limb interface may also cause decreases in venous return and reduce lymphatic drainage, which can be detrimental to amputees with compromised vascular systems (2, 6, 7). Transverse plane moments applied by the prosthetic socket to the residual limb, can peak during turning maneuvers and exacerbate the problem (8, 9). If turning maneuvers were uncommon, little emphasis on this problem would be warranted. However, turning maneuvers comprise a sizeable fraction of the steps taken during typical daily activities (10–12). Amputees also experience back pain at a higher rate than the general population (13), resulting in part from an asymmetric gait (14). Undesirable transverse plane moments may be a factor in asymmetrical, compensatory gait.

The need to ameliorate transverse plane moments between the residual limb and socket was recognized as early as 1947 by Eberhart (15) who wrote that transverse plane motions and their frictional effects “are a major source of discomfort and the chief cause of dissolution of the skin.” Three decades later, Lamoureux and Radcliffe (16) presented a prosthesis with an elastomeric spring allowing axial rotation in between the ankle and the socket and found that its use provided “dramatic relief of skin abrasions and epidermoid cysts in some cases”. In addition to reducing the transverse plane moment, they also reported improved gait symmetry. Today, transverse plane rotation adapters as a standalone device are commercially-available and can increase transverse plane rotation and decrease transverse plane moments (8, 17). They can also reduce the energy consumption of unilateral amputees at walking speeds above normal (18). This transverse rotation function can also be found in other commercially available devices such as shock absorbing pylons and multiaxial feet.

These observations suggest that prescription of transverse plane rotation adapters may lead to greater mobility for lower limb amputees. However, their use is not widespread and if excessively compliant, may reduce gait stability (19). Cost, weight, prosthesis build height, and the inability for the user to adjust the stiffness may all play a role in their lack of adoption, but it may also be that the transverse plane rotation is not coupled with the sagittal plane. With these devices, motion only occurs in the transverse plane when a transverse plane torque is applied. In contrast, the intact foot and ankle contain a complex set of joints that allows rotation in all three planes, and some are coupled together (20, 21), meaning rotation in one plane induces motion in another. In particular, the axis of rotation of the talo-crural joint during ankle flexion is inclined downwards and laterally relative to horizontal, and the rotation ranges from 10 to 26 degrees among individuals (22). The rotation about this inclined axis couples plantar- and dorsi-flexion motion with external and internal rotation of the shank relative to the foot, respectively (23). This sagittal:transverse ankle angle coupling is not replicated in prosthetic feet and ankles.

Ambulatory individuals with a lower limb amputation take thousands of steps on their prosthesis each day (24, 25) and none feature coupled motion between the sagittal- and transverse planes. The absence of this natural coupling may be related to the high incidence of residual limb soft tissue injuries (6, 7), the need for compensatory gait (14), and overall dissatisfaction with their prostheses (26, 27).

The purpose of this study was to determine if a sagittal:transverse ankle angle coupling ratio exists that minimizes the peak transverse plane moment (socket torque), normalized to body mass, during prosthetic limb stance. A novel, torsionally active prosthesis (TAP) was used to couple sagittal and transverse plane motions using a 60-watt motor (28). An embedded controller generated transverse plane rotation trajectories proportional to sagittal plane ankle angles corresponding to sagittal:transverse coupling ratios of 1:0 (rigid coupling analogous to the standard-of-care), 6:1, 4:1, 3:1, and 2:1. Thus, for a 6:1 coupling ratio, if a subject generates a sagittal plane motion of six degrees with their prosthesis, the TAP will generate a transverse plane motion of one degree. Individuals with unilateral transtibial amputation walked in a straight line and in both directions around a circle with the TAP set at different coupling ratios (blinded and randomized). The primary outcome was the peak transverse plane moment, normalized to body mass, during prosthetic limb stance. Secondary outcomes included gait biomechanic metrics and a measure of satisfaction.

## 2 Materials and methods

To discover the influence of coupled motion on the gait biomechanics of individuals with lower limb amputation, we built a novel, Torsionally Active Prosthesis (TAP) whose transverse plane motion (driven by a motor) could be controlled in proportion to sagittal plane motion (driven by the wearer) using real-time feedback and an on-board microcontroller. This novel prosthesis was then fitted to participants who provided informed consent to an Institutional Review Board approved human subjects experiment.

### 2.1 Torsionally active prosthesis

**Figure 1:**
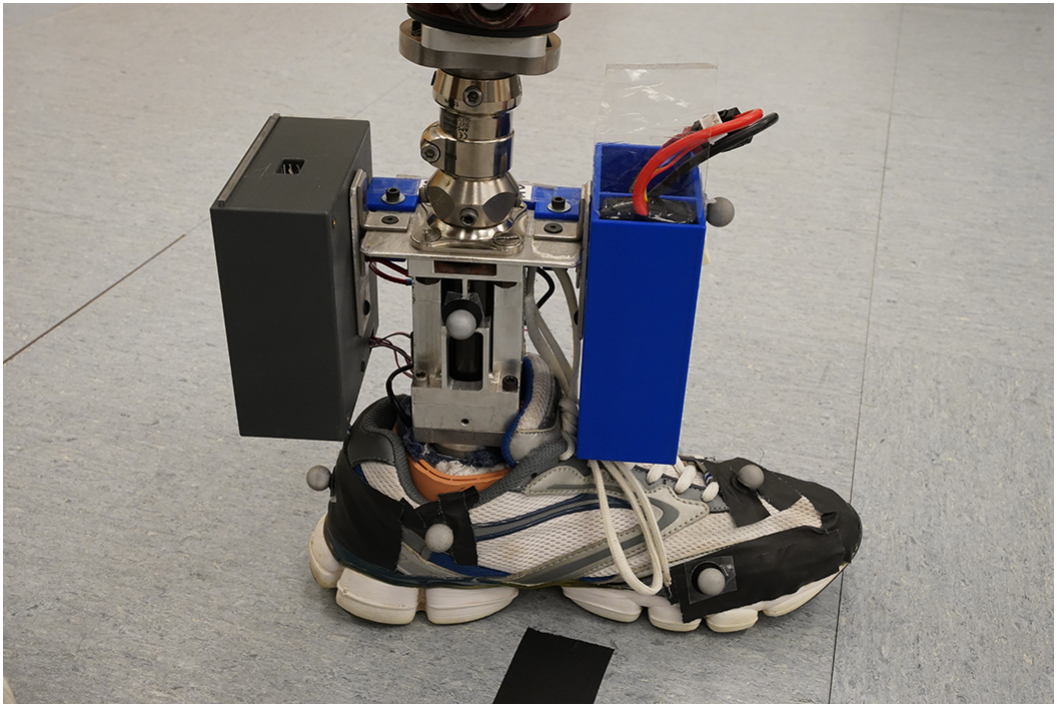
The second-generation TAP.

**Figure 2:**
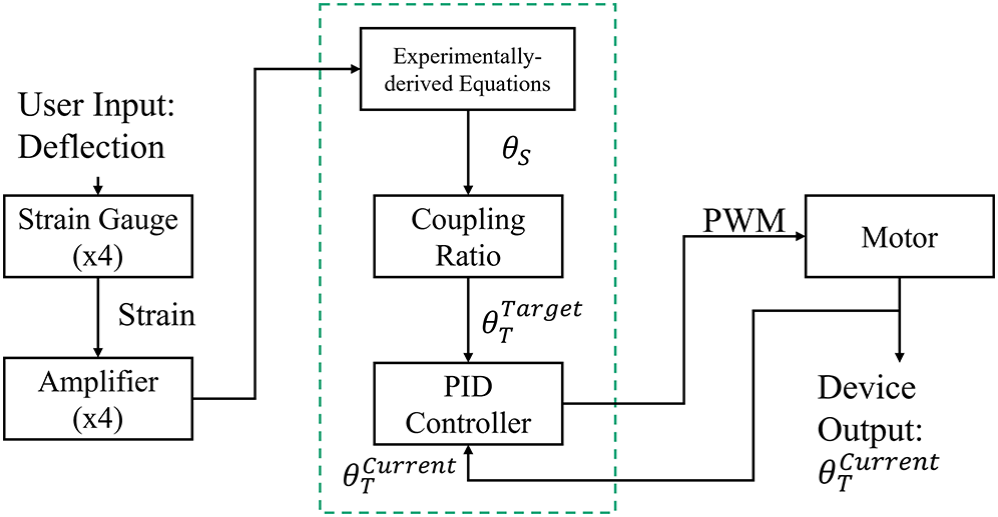
TAP system architecture. Proportional integral derivative (PID), pulse width modulation (PWM), sagittal plane angle (*θ_S_*), target transverse plane angle (*θ_T_*^Target^), actual transverse plane angle (*θ_T_*^Current^).

The TAP is based on a series elastic actuator composed of a 60-watt brushed, direct current, battery powered motor (RE30, Maxon Precision Motors, San Mateo, CA) in series with a 100:1 harmonic drive transmission (CSF14-2XH-F, Harmonic Drive, Hauppauge, NY), and an aluminum motor housing that acts as both a torsion spring and a torque transducer [first-generation TAP is described in (28)]. The second-generation TAP (see Figure 1) replaced an obsolete microcontroller with a 32-bit, 180 MHz microcontroller (Teensy 3.6, PJRC, Sherwood, OR) and strain gages to provide a robust estimate of the sagittal plane ankle angle. Body weight load tests were performed on different stiffness category prosthetic feet (Vari-Flex Low Profile, Össur, Reykjavik, Iceland) with strain gages mounted on the forefoot keel and heel keel. Individuals are prescribed feet with a specific stiffness category based on their body weight and activity level. Data from these non-human subject tests were used to obtain prosthetic foot category-dependent transfer functions to convert measured strain to an estimation of the sagittal plane ankle angle. The transverse plane angle was calculated using a magnetic encoder (Encoder MR, Type L, Maxon Precision Motors) that measured motor position. Using the experimentally derived transfer functions (one for each category stiffness prosthetic foot and motor position), the system software (see Figure 2) calculates the target transverse plane rotation trajectories corresponding to sagittal:transverse coupling ratios of 1:0 (rigid), 6:1, 4:1, 3:1, and 2:1 (the independent variable), which are then used in a proportional-integral-derivative (PID) controller tuned by a combination of Ziegler-Nichols (29) and manual tuning heuristics. The PID controller provides motor inputs used to achieve the desired coupling ratio during ambulation. The controller operated at a 1 kHz loop rate. A magnetic encoder (Encoder MR, Type L, Maxon Precision Motors, San Mateo, CA) with 1,024 counts per turn was used to calculate transverse plane rotation. Motor current was used to calculate transverse plane moments normalized to body mass. Data was logged on to a micro-SD card at a sampling rate of 100 Hz.

To power the TAP, an on-board 11.1-volt 3-cell lithium polymer battery (20C 4,000 mA, Venom, Rathdrum, ID) allowed 60–90 min of operation. At 2.9 kg, the ready-to-test configuration (including shoe) was not expected to influence oxygen consumption, heart rate, or gait efficiency (30–32). The TAP can meet the operational requirements (<29 Nm transverse plane torque) of straight and circle walking activities (8, 33) of a 75th percentile male adult (∼100 kg) (34) who can accommodate a prosthesis with a minimum build height of 22 cm.

**Figure 3:**
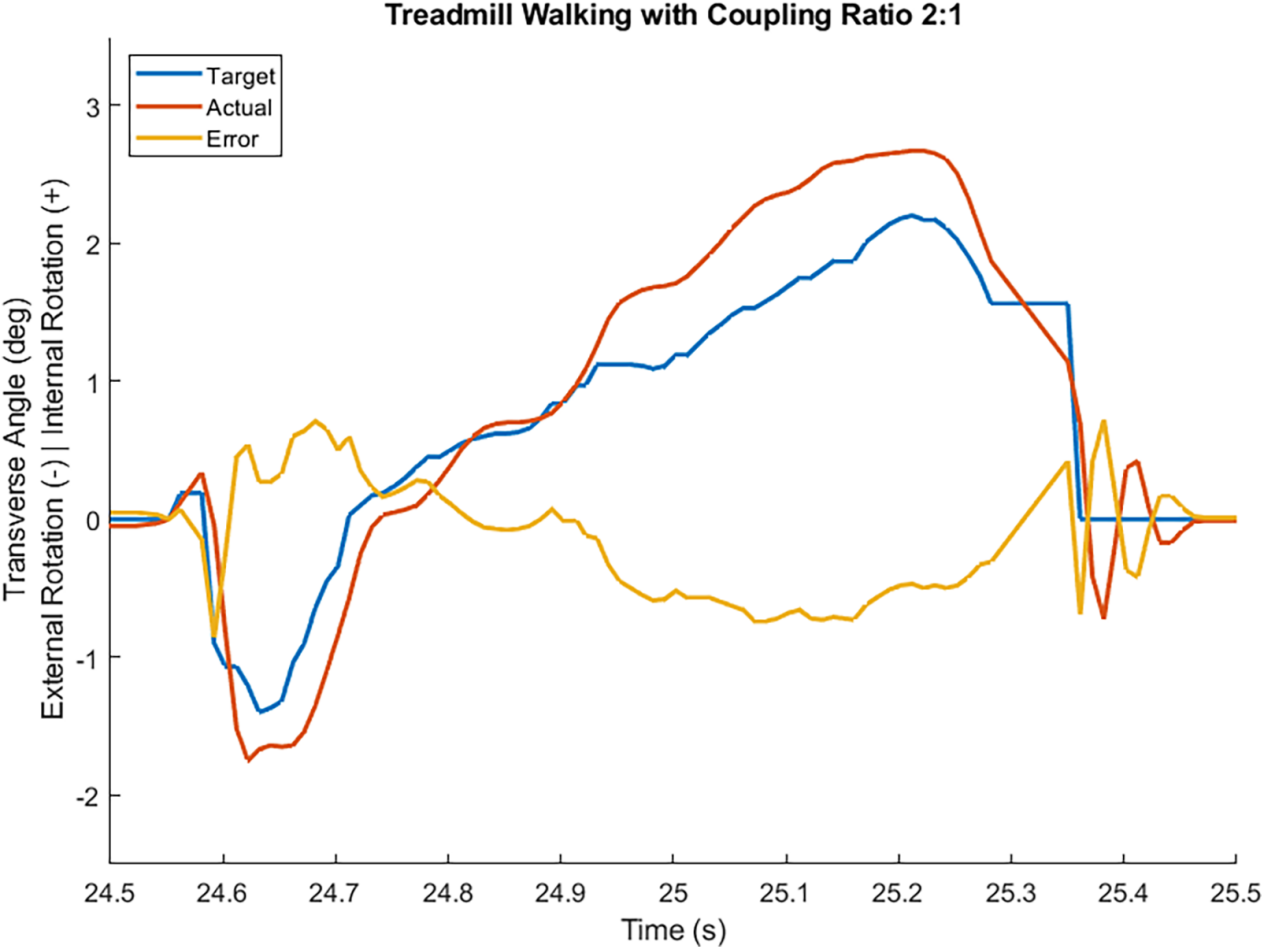
Transverse plane angle target, feedback driven actual response, and error while an individual without an amputation walked at self-selected speed wearing a boot cast arrangement in a straight line with coupling ratio 2:1. Heel contact occurred at 24.55 s and toe off occurred at 25.36 s.

**Figure 4:**
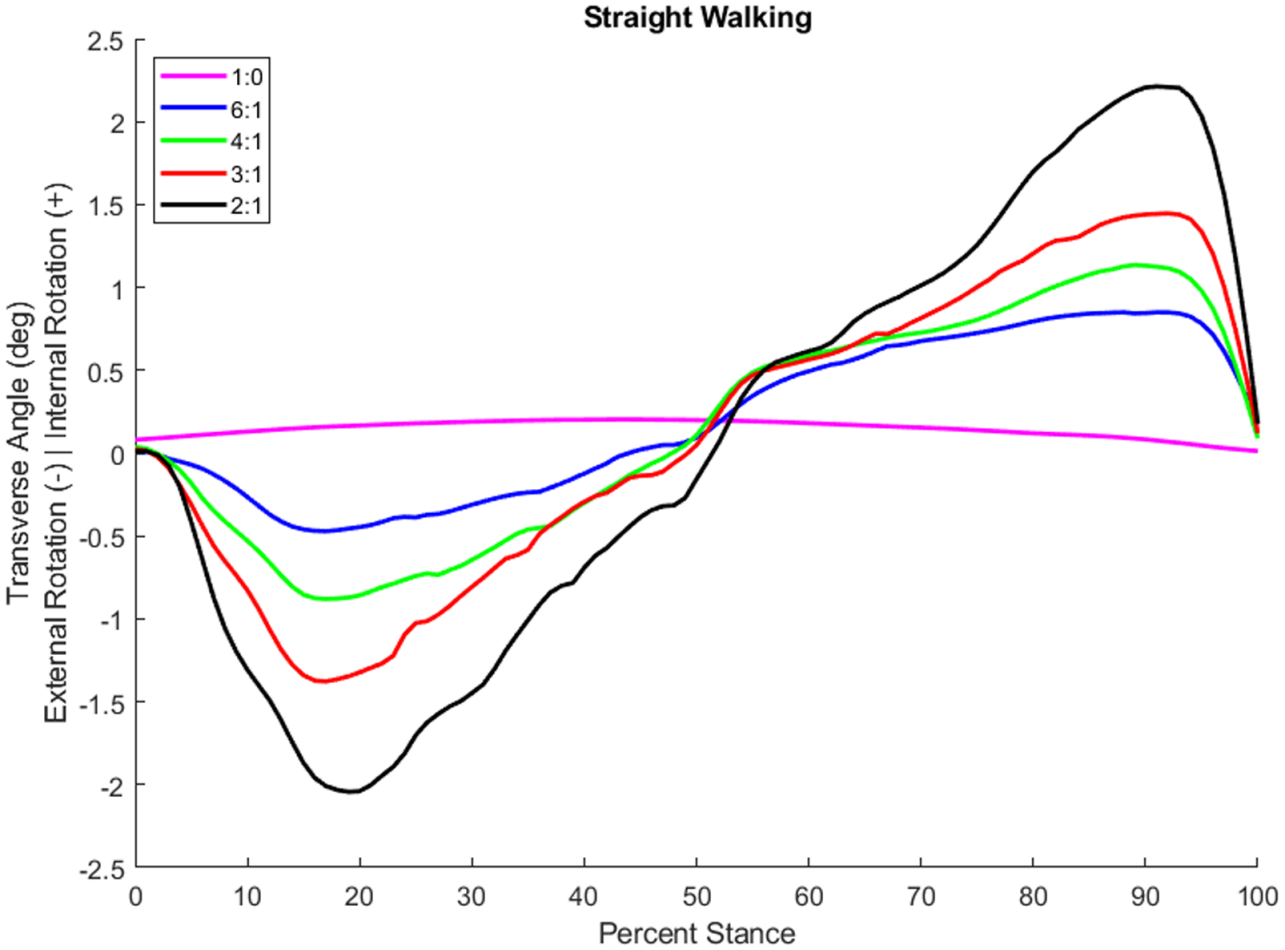
Transverse plane rotation angle during stance of an individual with a transtibial amputation while walking at self-selected speed in a straight line with different coupling ratios.

System operation tests using a boot cast arrangement boot cast arrangement enabling individuals without amputation to walk using the TAP over the range of coupling ratios. The most challenging condition is the 2:1 sagittal:transverse coupling ratio as it demands the largest transverse plane rotation. During early stance, the difference between the target and feedback driven response was very small and the performance profile closely followed the target (see Figure 3). During late stance, the difference between the target and feedback driven response remains relatively small and the performance profile follows the target but not as closely (see Figure 3). This late stance drop in performance is to be expected as the load on the motor is much greater. The error, the difference between the target (setpoint) and the feedback driven response, during a complete gait cycle was only 0.269° RMS. Another key metric of performance is the amount of current required during system operation. Large currents place much greater performance requirements on the electronic components and battery. Peak current was less than 30 amperes and averaged 7.5 amperes over the gait cycle. The range of transverse angles achieved varied by coupling ratio (see Figure 4 for a representative test subject result).

### 2.2 Human subject experiment

#### 2.2.1 Participants

Eleven males with unilateral transtibial amputation (age: 53 ± 15 years, height: 1.76 ± 0.06 m, mass: 92 ± 11 kg, etiology: 8 trauma, 2 diabetic, 1 infection; see Table 1 for as-prescribed prosthetic prescription) participated. All were free of contractures, had been fitted with a prosthesis and had used a prosthesis for at least six months, wore their prosthesis at least 4 h per day, and were moderately active by self-report suggesting all were capable of community ambulation. Each provided informed consent approved by the governing Institutional Review Board.

**Table 1:** Sample demographics and as-prescribed prosthetic prescription. patellar-tendon-bearing (PTB), total surface bearing (TSB).

Subject	Age (years)	Height (m)	Mass (kg)	Etiology	Prescribed Foot	Liner	Socket
1	58	1.68	84	Trauma	College Park Tru Step	ALPS 26	Hybrid PTB
2	55	1.75	89	Infection	Össur Proflex XC Torsion	Willowood Alpha Silicone	TSB
3	70	1.86	83	Trauma	Fillauer All Pro	Willowood Alpha Classic	Hybrid PTB
4	57	1.78	86	Trauma	Cheetah Explorer	Össur Iceross Comfort	TSB
5	72	1.75	86	Diabetes	Echelon VT	Willowood Alpha Classic	TSB
6	31	1.84	100	Trauma	College Park Tactical	Össur Iceross	TSB Boa System
7	29	1.65	83	Trauma	Össur XC Torsion	Össur Iceross	Modified PTB
8	60	1.79	112	Trauma	Össur Pivot	Willowood Alpha Classic	TSB
9	70	1.79	83	Diabetes	College Park Tru Step	Willowood Alpha Classic	PTB
10	42	1.81	111	Trauma	IBEX Filauer	Össur Iceross Comfort	PTB
11	40	1.71	97	Trauma	Össur LP Proflex	Össur Iceross	TSB with adjustable posterior panel

#### 2.2.2 Study prosthesis

Each subject was fit with the TAP in series with a size and stiffness category appropriate Vari-Flex Low Profile prosthetic foot and foot cover by a certified prosthetist. The prosthetic pylon height was adjusted such that the build height of the study prosthesis was equivalent to the subject's as-prescribed prosthesis.

#### 2.2.3 Instrumentation

Data for biomechanic outcomes were measured with embedded force plates and a motion capture system. Eight force plates (BP400600, AMTI, Watertown, MA) mounted flush to the floor measured ground reaction forces (GRF) at 2,000 Hz and were filtered with a bidirectional Butterworth filter with a 25 Hz cutoff. All subjects were provided with tight fitting spandex shorts and shirts to wear during data collection. The same researcher placed 14 mm reflective tracking markers on each subject using Vicon's standard Plug-in-Gait marker set, with additional markers placed bilaterally on the medial elbow, medial knee epicondyle, medial malleolus, tibial tuberosity, fibular head, and first and fifth metatarsal heads. Clusters of four markers were also placed bilaterally on the upper arms and thighs. The markers on the prosthetic limb mirrored the intact limb. TAP-specific markers were added to the anterior and posterior faces of the device as well as the medial and lateral base of the motor housing. A 16-camera motion capture system (Vantage V8, Vicon Motion Systems, Oxford, UK) recorded marker trajectories at 100 Hz which were filtered with a bidirectional Butterworth filter with a 6 Hz cutoff.

Satisfaction for each condition was captured with a single score on a 0–10 scale. Zero represented the most uncomfortable socket fit the subject could imagine, and ten represented the most comfortable socket fit.

#### 2.2.4 Protocol

Self-selected walking speeds (SSWS) were calculated from the mean of three trials while the subjects wore their as-prescribed prosthesis to walk at their own pace 20 m in a straight (ST) line and while walking around a 2 m diameter circle marked with a dashed line on the floor with their prosthesis on the inside (PI) and outside (PO) of the circle. Subject height, body mass, and demographics were also recorded. The TAP was then fit and aligned by a licensed and certified prosthetist using standard clinical procedures. The pylon length was adjusted to accommodate the build height of the TAP as needed. Each subject wore their as-prescribed socket and suspension system except for one whose foot was mounted posteriorly directly to the socket. For this subject, a duplicate socket was made with a conventional pyramid adapter with which to mount the TAP. Although there is no consensus on how much accommodation time is necessary for acclimation to a new prosthetic foot (35), we allowed each subject at least 15 min to walk straight and around the 2 m circle with the TAP to learn how each coupling ratio performed and felt.

Subjects were block randomized to the order in which they walked ST, PI, and PO. Sagittal:transverse coupling ratios of 1:0, 6:1, 4:1, 3:1, and 2:1 were also block randomized and blinded to the subject. At least ten trials each of ST, PI, and PO were performed with a minimum of two trials at each coupling ratio. Acceptable trials were within ± 10 percent of their self-selected walking speed and had at least one single limb foot-ground contact wholly on a force plate for each limb. However, while wearing the TAP, some subjects (*n* = 5) consistently had difficulty walking at their SSWS previously measured while wearing their as-prescribed prosthesis. For these subjects, their SSWS was recalculated while wearing the TAP and walking at their own pace for 6 m in a ST line and while walking around a 2 m diameter circle as previously described. After each set of trials at a specific coupling ratio, the subject was asked to rate their satisfaction. Rest breaks were provided as needed. If all planned trials could not be completed within 4 h, the subject returned for a second visit after at least one overnight rest period.

### 2.3 Analysis

The marker trajectories and GRFs were processed in Visual 3D (C-Motion, Boyds, MD) to calculate gait kinematics, kinetics, and gait event timings. Knee and hip joint angles and powers were calculated using a 15-segment whole body model (head, torso, Visual 3D Composite pelvis, and bilateral upper arm, forearm, hand, thigh, shank, and foot). Prosthetic ankle power was calculated using the unified deformable segment model (36). The coordinate systems were transformed to the subject's torso coordinate system to maintain alignment with the direction of progression. Each segment's mass was estimated as a percentage of whole-body mass (37), and the inertial properties and center of mass positions were based on geometric approximations calculated in Visual 3D. The prosthetic shank mass was reduced to 35% of the intact shank, and the prosthetic CoM location was moved 35% closer to the knee joint (38). All GRFs were normalized by subject body mass (kg). Initial heel contact and toe-off events were automatically detected based on force plate loading threshold of 25 N and kinematic pattern recognition. These events were also inspected visually and corrected if needed.

The primary outcome was the peak transverse plane moment, normalized to body mass, during prosthetic limb stance. To discover if varying the coupling ratio influenced the subject's gait or their satisfaction with their prosthesis, secondary outcomes were calculated. Discretized gait biomechanic metrics included the intact and prosthetic hip and knee power during push-off [known as H3 and K3, respectively (39)], and the prosthetic ankle power during push-off. Kinematic metrics included peak hip extension angle during pre-swing and peak knee flexion during weight acceptance. The vertical and anterior-posterior GRF during weight acceptance were also analyzed. All outcomes including satisfaction were aggregated with project specific software (MathWorks, Natick, MA).

Linear mixed effects regression was used to test for an association between each outcome (dependent variable) by coupling ratio. Coupling ratio was the independent fixed effect (modeled as categorical using 4 dummy variables). Study participant and study participant by coupling ratio interaction were random effects. To address the variability in outcome variance among participants, maximum penalized likelihood estimation was used (40). Hypothesis testing for the association between outcome and coupling ratio was carried out using conditional F-tests with degrees of freedom estimated using the Kenward-Roger method. Pairwise hypothesis testing was carried out with adjustments for multiple comparisons using Tukey's method. Results are summarized as outcome means (±standard error) by coupling ratio, and pairwise mean differences in outcome by coupling ratio category accompanied by standard errors and 95% confidence intervals (CI). Analyses were carried out using R 4.2.1 (41), and packages tidyverse, lme4, blme and emmeans (40, 42–44). Statistical analyses on satisfaction results were not performed due to the small sample size and the higher expected variances of qualitative data.

## 3 Results

The subjects' ST, PI, and PO SSWS were 1.24 ± 0.19 m/s (mean ± standard deviation), 0.44 ± 0.08 m/s, and 0.42 ± 0.07 m/s, respectively. Eleven subjects completed all trials walking ST and PI. A device malfunction prevented one subject from completing the PO trials.

**Figure 5:**
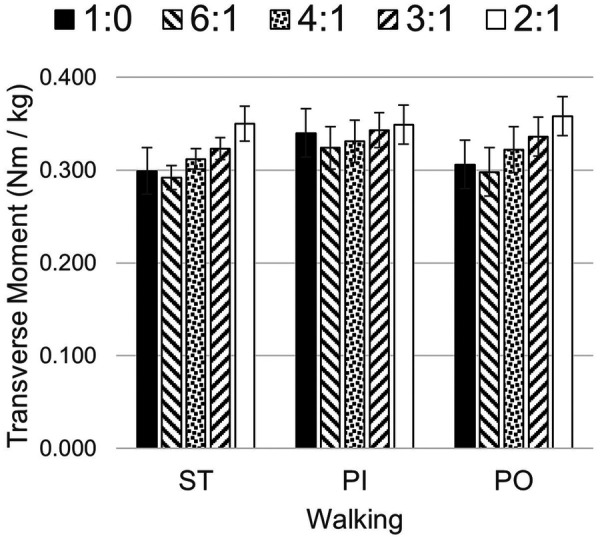
Transverse plane moments while walking straight (ST) and around a 2 m diameter circle with the prosthesis on the inside (PI) and on the outside (PO) at five different sagittal:transverse coupling ratios.

There were significant differences in the mean maximum transverse plane moments while ST and PI walking (see Table 2). The general pattern suggest a quadratic relationship between transverse plane moments and the sagittal:transverse ankle angle coupling ratio with a minimum at 6:1 (see Figure 5). Coupling ratios greater than 6:1 (i.e., 4:1, 3:1, and 2:1) appear to increase the transverse plane moment when compared to the rigid condition (1:0). However, only the 6:1 vs. 3:1 and the 6:1 vs. 2:1 coupling ratios during ST and PO walking were statistically different (*p* < .05) in pairwise comparisons (see Table 3). The general pattern also suggests the transverse plane moments were lowest during ST and highest PI walking (see Figure 5).

**Table 2:** Mean (±SE) maximum transverse plane moment normalized to body mass (Nm/kg) while walking straight (ST) and around a 2 m diameter circle with the prosthesis on the inside (PI) and on the outside (PO) at five different sagittal:transverse coupling ratios.

	Sagittal:transverse coupling ratio					
	1:0	6:1	4:1	3:1	2:1	*p*-value
ST	0.299 ± 0.025	0.292 ± 0.013	0.312 ± 0.011	0.323 ± 0.012	0.350 ± 0.019	**0** **.** **029**
PI	0.340 ± 0.026	0.324 ± 0.023	0.331 ± 0.023	0.343 ± 0.019	0.349 ± 0.021	0.340
PO	0.306 ± 0.026	0.298 ± 0.026	0.322 ± 0.025	0.336 ± 0.021	0.358 ± 0.021	**0**.**040**

Bold font indicates a statistically significant difference at *p* < 0.05.

**Table 3:** Mean pairwise difference (± SE), 95% CIs and *p*-values in maximum transverse plane moment normalized to body mass (Nm/kg) among sagittal:transverse coupling ratios while walking straight (ST) and around a 2 m diameter circle with the prosthesis on the inside (PI) and on the outside (PO).

Coupling ratios	ST	PI	PO
1:0–6:1	0.006 ± 0.018 (−0.054, 0.067) 1	0.017 ± 0.014 (−0.031, 0.064) 0.78	0.008 ± 0.015 (−0.044, 0.060) 0.99
1:0–4:1	−0.013 ± 0.018 (−0.072, 0.045) 0.94	0.009 ± 0.015 (−0.041, 0.060) 0.97	−0.016 ± 0.015 (−0.068, 0.036) 0.83
1:0–3:1	−0.024 ± 0.021 (−0.092, 0.044) 0.77	−0.003 ± 0.020 (−0.068, 0.063) 1	−0.031 ± 0.015 (−0.083, 0.021) 0.33
1:0–2:1	−0.051 ± 0.023 (−0.128, 0.025) 0.26	−0.008 ± 0.017 (−0.064, 0.048) 0.99	−0.052 ± 0.021 (−0.122, 0.018) 0.17
6:1–4:1	−0.02 ± 0.009 (−0.048, 0.009) 0.24	−0.007 ± 0.009 (−0.035, 0.021) 0.91	−0.024 ± 0.009 (−0.053, 0.006) 0.14
6:1–3:1	**−0.031 ± 0.009** **(−0.061, 0)** **0.047**	−0.019 ± 0.012 (−0.06, 0.021) 0.55	**−0.038 ± 0.011** **(−0.074, −0.002)** **0.036**
6:1–2:1	**−0.058 ± 0.014** **(−0.105, −0.011)** **0.016**	−0.025 ± 0.014 (−0.07, 0.021) 0.42	**−0.06 ± 0.016** **(−0.113, −0.006)** **0.028**
4:1–3:1	−0.011 ± 0.008 (−0.038, 0.016) 0.69	−0.012 ± 0.011 (−0.048, 0.024) 0.8	−0.015 ± 0.01 (−0.048, 0.018) 0.57
4:1–2:1	−0.038 ± 0.014 (−0.083, 0.007) 0.11	−0.018 ± 0.009 (−0.049, 0.014) 0.39	−0.036 ± 0.014 (−0.082, 0.01) 0.14
3:1–2:1	−0.027 ± 0.010 (−0.06, 0.006) 0.13	−0.006 ± 0.011 (−0.043, 0.032) 0.99	−0.021 ± 0.010 (−0.055, 0.013) 0.29

Bold font indicates a statistically significant difference at *p* < 0.05.

Biomechanical outcomes did not exhibit any statistical differences across coupling ratios during ST, PI, and PO walking (see Table 4).

**Table 4:** Mean (± SE) kinetic and kinematic biomechanical outcomes by sagittal:transverse plane coupling ratio while walking straight (ST) and around a 2 m diameter circle with the prosthesis on the inside (PI) and on the outside (PO).

	Sagittal:transverse coupling ratio					
	1:0	6:1	4:1	3:1	2:1	*p*-value
Peak Hip Power (H3) Intact Limb (W/kg) during push-off						
ST	0.88 ± 0.12	0.86 ± 0.13	0.84 ± 0.12	0.87 ± 0.13	0.92 ± 0.14	0.49
PI	0.75 ± 0.11	0.7 ± 0.1	0.79 ± 0.13	0.77 ± 0.11	0.71 ± 0.11	0.45
PO	0.47 ± 0.05	0.49 ± 0.07	0.48 ± 0.05	0.45 ± 0.05	0.5 ± 0.06	0.85
Peak Hip Power (H3) Prosthetic Limb (W/kg) during push-off						
ST	0.55 ± 0.09	0.55 ± 0.08	0.53 ± 0.09	0.59 ± 0.08	0.56 ± 0.09	0.28
PI	0.6 ± 0.07	0.62 ± 0.07	0.63 ± 0.07	0.65 ± 0.08	0.61 ± 0.08	0.69
PO	0.34 ± 0.06	0.34 ± 0.05	0.35 ± 0.07	0.33 ± 0.05	0.32 ± 0.05	0.95
Peak Knee Power (K3) Intact Limb (W/kg) during push-off						
ST	−1.3 ± 0.15	−1.25 ± 0.16	−1.21 ± 0.16	−1.27 ± 0.18	−1.26 ± 0.17	0.59
PI	−0.84 ± 0.11	−0.81 ± 0.1	−0.79 ± 0.09	−0.78 ± 0.11	−0.88 ± 0.12	0.68
PO	−1.12 ± 0.17	−1.1 ± 0.18	−1.13 ± 0.22	−1.09 ± 0.16	−1.12 ± 0.2	1.00
Peak Knee Power (K3) Prosthetic Limb (W/kg) during push-off						
ST	−1.24 ± 0.19	−1.16 ± 0.19	−1.17 ± 0.16	−1.23 ± 0.16	−1.2 ± 0.17	0.82
PI	−0.64 ± 0.09	−0.69 ± 0.1	−0.73 ± 0.11	−0.7 ± 0.1	−0.66 ± 0.1	0.55
PO	−1.23 ± 0.12	−1.22 ± 0.18	−1.3 ± 0.13	−1.3 ± 0.19	−1.24 ± 0.13	0.87
Peak Prosthetic Ankle Power (W/kg) during push-off						
ST	−0.15 ± 0.03	−0.15 ± 0.03	−0.15 ± 0.03	−0.16 ± 0.03	−0.16 ± 0.03	0.70
PI	−0.16 ± 0.04	−0.16 ± 0.04	−0.17 ± 0.04	−0.17 ± 0.04	−0.17 ± 0.05	0.78
PO	−0.15 ± 0.03	−0.14 ± 0.04	−0.16 ± 0.03	−0.13 ± 0.03	−0.15 ± 0.03	0.16
Peak Extension Hip Angle Intact Limb (°) during push-off						
ST	−18.3 ± 3.4	−17.9 ± 3.2	−18 ± 3.4	−18.5 ± 3.2	−18.5 ± 3.2	0.55
PI	−10.3 ± 3.7	−9.8 ± 3.6	−10 ± 3.7	−11.2 ± 3.7	−10.9 ± 3.8	0.50
PO	−10.3 ± 3.7	−9.9 ± 3.8	−10.5 ± 3.7	−10.1 ± 3.6	−10.1 ± 3.7	0.72
Peak Hip Extension Angle Prosthetic Limb (°) during push-off						
ST	−13.7 ± 3.3	−13.5 ± 3.1	−13.7 ± 3.3	−13.3 ± 3.4	−13.3 ± 3.4	0.70
PI	−6.6 ± 3.7	−6.4 ± 3.9	−7.1 ± 3.4	−6.9 ± 3.6	−6.9 ± 3.6	0.96
PO	−7.4 ± 3.3	−7.8 ± 3.4	−8 ± 3.2	−7.4 ± 3.5	−7.9 ± 3.4	0.75
Peak Knee Angle Intact Limb (°) during weight acceptance						
ST	12.8 ± 2.7	12.8 ± 2.7	12.9 ± 3	13.2 ± 2.6	12.7 ± 2.6	0.92
PI	8.5 ± 2.4	8.7 ± 2.4	8.9 ± 2.4	8.6 ± 2.4	8.4 ± 2.4	0.85
PO	12.4 ± 2.6	11.6 ± 2.4	12.6 ± 2.1	12.4 ± 2.6	11.9 ± 2.6	0.80
Peak Knee Angle Prosthetic Limb (°) during weight acceptance						
ST	7.8 ± 3.5	8.7 ± 3.3	7.4 ± 3.4	7.9 ± 3.6	8.8 ± 3.8	0.59
PI	8.5 ± 3.3	8 ± 3	8.4 ± 3.3	8.9 ± 2.9	8.8 ± 2.9	0.90
PO	9 ± 3.2	9.6 ± 2.9	9.8 ± 2.9	10.4 ± 2.8	9.6 ± 2.8	0.44
Vertical Ground Reaction Force (N/BW) during weight acceptance						
ST	1.11 ± 0.04	1.08 ± 0.03	1.08 ± 0.04	1.07 ± 0.05	1.1 ± 0.04	0.61
PI	1 ± 0.03	1 ± 0.03	1.01 ± 0.02	1.02 ± 0.03	1.02 ± 0.02	0.85
PO	1.03 ± 0.04	1.02 ± 0.04	1.03 ± 0.04	0.98 ± 0.04	1.03 ± 0.05	0.59
Anterior (braking) Ground Reaction Force (N/BW) during weight acceptance						
ST	−0.15 ± 0.03	−0.15 ± 0.03	−0.15 ± 0.03	−0.16 ± 0.03	−0.16 ± 0.03	0.70
PI	−0.16 ± 0.04	−0.16 ± 0.04	−0.17 ± 0.04	−0.17 ± 0.04	−0.17 ± 0.05	0.78
PO	−0.15 ± 0.03	−0.14 ± 0.04	−0.16 ± 0.03	−0.13 ± 0.03	−0.15 ± 0.03	0.16

Satisfaction results did not appear to exhibit any general patterns other than participants felt their prosthesis was comfortable as mean scores for the different coupling ratios ranged from a low of 7.1 to a high of 8.5 (see Table 5). Multiple subjects failed to discern any differences by coupling ratio. During ST walking, 4 of the 11 subjects gave the same score for each coupling ratios. During PI walking, only 2 of 11 subjects felt no differences across coupling ratios, but during PO walking 5 of 10 who completed all trials could not distinguish any differences in satisfaction.

**Table 5:** Mean (± SD) satisfaction scores on a 0–10 scale where 0 represents the most uncomfortable socket fit the subject could imagine, and 10 represents the most comfortable socket fit.

	Sagittal:transverse coupling ratio				
	1:0	6:1	4:1	3:1	2:1
ST	8.0 ± 1.3	8.2 ± 1.3	8.0 ± 1.0	7.7 ± 1.2	8.5 ± 1.0
PI	7.5 ± 1.3	7.9 ± 1.1	7.1 ± 1.7	7.5 ± 0.7	8.1 ± 1.3
PO	7.7 ± 1.7	7.9 ± 1.2	7.4 ± 1.3	7.8 ± 1.1	7.7 ± 1.5

## 4 Discussion

The study investigated the effects of varying the sagittal:transverse coupling ratio on individuals with a unilateral transtibial amputation while they walked straight and in both directions around a 2 m circle.

### 4.1 Interpretation

The ST SSWS for the participants in this study is comparable to the speeds reported for an identical task and similar population [1.24 ± 0.19 m/s vs. 1.19 ± 0.16 m/s (33), respectively]. However, the turning SSWS of the current population was slower than that previously reported [PI: 0.44 ± 0.08 m/s and PO: 0.42 ± 0.07 m/s m/s vs. mean of both directions: 0.88 ± 0.10 m/s (33), respectively]. The difference could be due to the greater mass of the TAP than the as-prescribed prostheses worn in (33).

The mean maximum transverse plane moments general pattern (see Figure 5) suggests a coupling ratio of 6:1 may reduce transverse plane moments during straight and circle walking, while a coupling ratio of 2:1 or 3:1 may increase them. The slower PI and PO speeds of the participants in this study may have reduced the magnitude of these moments (45). Subjects who walk faster around a circle may exhibit larger transverse plane moments. The transverse plane moments reported here are in general somewhat larger than those observed by subjects wearing a rigid pylon or a commercially-available transverse plane rotation adapter (8). The higher moments may be due to the greater mass of the TAP.

The joint powers (hip, knee, and prosthetic ankle) and kinematics (hip angle) during push-off and the kinematics (hip angle) during pre-swing were not affected by the coupling ratio. This suggests that the coupling ratios explored in this study do not adversely affect propulsion. The kinematics (knee angle) and GRFs (vertical and anterior-posterior) during weight acceptance were also not affected by the coupling ratio. This suggests the coupling ratios explored in this study do not adversely affect body support.

The satisfaction ratings were also not influenced by the coupling ratio. Likert scales like the one used in this study can allow for a range of responses from one extreme to the other as well as no opinion. The results here suggest the subjects were comfortable with their prosthesis and the coupling ratio had no discernible effect. More advanced methods may need to be adapted for use with the TAP to explore this issue (46).

### 4.2 Implications

For individuals with lower limb amputation who are capable of locomotion, their clinician must choose among several hundred available prosthetic feet when prescribing a prosthesis. While these products have many different distinguishing features, none mimic the coupled motion exhibited by the natural limb. The results of this study suggest a coupling ratio exists that minimizes transverse plane moments without adversely affecting key gait metrics or satisfaction with their prosthesis. The target population for this device is the limited and unlimited community ambulator. Household ambulators may have challenges associated with balance and the coupled transverse plane motion could potentially induce instability in these individuals. At the other end of the spectrum, the athletic ambulator with high impact loads and large sagittal plane motions might generate excessive coupled transverse plane motions which could cause skin irritation or injury arising from high shear stresses.

### 4.3 Limitations

Limitations of this research include a small sample population (*n* = 11), a short acclimation period to a novel intervention (15 min), a heavier intervention than the participant's as-prescribed prosthesis, and a limited selection of tested coupling ratios (five). A larger sample population might produce additional results with statistical significance. A longer acclimation period might result in subjects walking faster around the 2 m circle. A longer acclimation period might also enable the subjects to be more nuanced in their opinions resulting in observable differences in satisfaction scores between conditions. The TAP is approximately three times as heavy as a conventional prosthetic foot. While prescription of a conventional transverse plane rotation adapter would reduce this difference, a heavier study intervention might bias satisfaction ratings. Finally, while this study explored five different coupling ratios, the TAP could be programmed to explore a range more closely centered on the 6:1 coupling ratio.

### 4.4 Future work

The clinical significance of this research lies in the development of a passive (i.e., not mechatronic) version of the TAP and measure its safety and effectiveness in a long-duration, field-based clinical trial. A clinical trial comparing a passive version of the TAP to a rigid pylon and a transverse plane rotation adapter would illuminate key differences.

## Data Availability

The raw data supporting the conclusions of this article will be made available by the authors, without undue reservation.
